# Participants’ Utilitarian Choice Is Influenced by Gamble Presentation and Age

**DOI:** 10.3390/bs14070536

**Published:** 2024-06-26

**Authors:** Joseph Teal, Petko Kusev, Siana Vukadinova, Rose Martin, Renata M. Heilman

**Affiliations:** 1 Behavioural Research Group, London South Bank University Business School, London South Bank University, London SE1 0AA, UK; kusevp@lsbu.ac.uk; 2 Faculty of Philosophy, Sofia University, 1504 Sofia, Bulgaria; ssvukadino@office365student.uni-sofia.bg; 3 Department of People and Organisations, Surrey Business School, University of Surrey, Guildford GU2 7XH, UK; r.k.martin@surrey.ac.uk; 4 Department of Psychology, Babeș–Bolyai University, 400015 Cluj-Napoca, Romania; renataheilman@psychology.ro

**Keywords:** utilitarian decision-making, gamble presentation, age, risk, probability, money

## Abstract

No prior behavioral science research has delved into the impact of gamble presentation (horizontal or vertical) on individuals’ utilitarian behavior, despite evidence suggesting that such choices can be influenced by comparing attributes like probability and money in gambles. This article addresses this gap by exploring the influence of gamble presentation on utilitarian behavior. A two-factor independent measures design was employed to explore the influence of the type of gamble presentation and age on participants’ utilitarian decision-making preferences. The findings showed a reduced likelihood of participants choosing the non-utilitarian gamble with vertically presented gambles compared to horizontal ones. Consequently, participants’ utilitarian behavior was influenced by between-gamble comparisons of available attributes, with utilitarian choices (e.g., choosing Gamble A) being more prevalent in vertical presentations due to a straightforward comparison on the probability attribute. Furthermore, the results also revealed that older participants take more time than their younger counterparts when making utilitarian errors. We attribute this to their abundant knowledge and experience. Future research should explore the comparative psychological processing used by participants in risky decision-making tasks.

## 1. Introduction

Behavioral science theorists assume that rational decision-makers are utility maximisers, who possess stable preferences, informed by consistent psychological processing (e.g., [1]). For example, when choosing between two gambles, people (i) integrate the probabilistic information (money and probability) within each gamble, (ii) compute the expected value of each gamble, and (iii) choose the gamble with the largest expected value. For instance, the expected value of a risky gamble with a 55% chance of winning GBP 0.50 is GBP 0.275; the financial outcome (GBP 0.50) is multiplied by the likelihood of the outcome occurring (55%). Similarly, the expected value of a risky gamble with a 15% chance of winning GBP 1.50 is GBP 0.225. Accordingly, theories of decision-making assume that people search for and process probabilistic attribute information ‘within gambles’.

In contrast to these assumptions, recent behavioral science research has revealed that people frequently violate normative assumptions. In particular, empirical studies indicate that people do not consistently compute expected value, frequently fail to maximize their utility, and have labile preferences [2,3,4]. Instead, people appear to construct their risk preferences ‘on the fly’ using flexible psychological processing. It is flexible because the psychological processes used to make decisions can be influenced by context and task [5], as well as other factors such as emotion [6,7], learning [8], and individual differences [6,9,10].

In this article, we seek to expand our previous research exploring the lability of human risk preferences, by exploring the possibility that gamble presentation (horizontal or vertical) can influence the construction of people’s risk preferences. For example, we will investigate how gamble presentation influences participants’ utilitarian behavior. This is a novel proposal that has not been explored in existing behavioral science research. Moreover, in this article, we will also explore the influence of age on people’s decision-making time (the time they take to make a decision).

### 1.1. Context and Decision-Making Tasks

Across countless empirical studies, behavioral science researchers have uncovered evidence which indicates that people’s risk preferences are strongly influenced by contextual factors. For example, Kusev et al. [4] revealed that an insurance decision is treated with greater risk-aversion than a structurally identical but de-contextualized economic gamble. Accordingly, the authors concluded that insurance decisions are influenced by the accessibility of risky events in memory; people’s memories of previous experiences ‘leak’ into subsequent precautionary choices. Similarly, Vlaev et al. [10] explored whether there was variability in participants’ risk preferences across several financial domains including, monetary gambles, insurance, investment, pension provision, mortgage buying, job salary change, and hazard losses. The results revealed that people’s risky attitudes and behaviors were highly context-sensitive. Moreover, in an influential study, Hertwig et al. [11] (see also [12]) revealed that when people make decisions from experience (sampling and memory) rather than decisions from description (described probabilistic options), they tend to underweight small probability events.

Furthermore, additional evidence for the influence of context on human risky behavior comes from Decision by Sampling [13], an influential model of relative judgment and decision-making. According to Stewart and colleagues’ Decision by Sampling model, people do not have stable and absolute subjective representations of objective attribute information (e.g., amounts of money). Instead, Stewart et al. proposed and argued that the subjective worth of an attribute value (e.g., an amount of money) is represented by its relative rank within the attribute (e.g., money) when compared against comparable attribute values (e.g., other amounts of money) sampled from experience (memory or the immediate context). As demonstrated by Ungemach et al. [14], this has implications for risky choice. In particular, during a choice between two gambles, Ungemach and colleagues demonstrated that people’s risky choice preferences were influenced by recently experienced prices (sampled monetary amounts). Specifically, when experienced prices were inside the range of the gambles’ prizes (between the prizes offered by both gambles), the gamble with the higher prize seemed more attractive. In contrast, when experienced prices were outside the range of the gambles’ prizes (below the lowest prize and above the largest prize), the gamble with the highest prize seemed less attractive (not much different from the gamble with the lowest prize).

A more recent empirical demonstration that contextual factors can influence risky choices comes from Kusev et al. [5]. In particular, Kusev and colleagues demonstrated that during repeated choice between a series of two economic gambles, one with a certain outcome and one with a probabilistic outcome, people’s choices were influenced by the distribution of the certain options around the expected values of the probabilistic options. Specifically, consistent with the predictions of Prospect Theory, when the gambles with certain outcomes were unevenly (logarithmically) distributed around the expected value of the probabilistic options, participants demonstrated a four-fold pattern of risk preferences: risk-aversion for high-probability gains and low-probability losses, and risk-seeking for low-probability gains and high-probability losses. In contrast, when the gambles with certain outcomes were evenly (linearly) spaced around the expected value of probabilistic options, participants were not influenced by probability levels, and therefore demonstrated a two-fold pattern: risk-seeking in the domain of loss and risk-aversion in the domain of gain.

Indeed, the notion that contextual factors can influence cognitive processing and people’s subsequent choice preferences has been explored empirically. For instance, across a series of experiments, Payne [15] demonstrated that information processing varies as a function of task complexity. In particular, while participants used normative processing with simple tasks, they were more likely to use simplifying heuristics (psychological shortcuts) as task complexity increased (see also [16]). In the context of risky choice, particular attention has been paid to examining the extent to which people’s choices are guided by ‘within gambles’ (i.e., computational processing) and ‘between gambles’ (i.e., comparison of gambles’ attribute values) processing of information (e.g., [17]). For example, in a study that employed eye-tracking technology, Fiedler and Glöckner [18] found that during choice between gambles, participants mostly engaged in computational processing and often selected the option with the highest expected value (see also [19]). In contrast, Russo and Dosher [20] found that during choice between risky gambles, people appear to compare gambles on each attribute individually (see also [21]). To account for the inconsistent findings, theorists have proposed and demonstrated that the ease at which gamble options can be computed [22] and whether gambles are played once (one-shot) or repeatedly [23] influence the psychological processing of information.

However, there are no published experimental studies that have explored the possibility that the presentation (i.e., horizontal or vertical) of gambles and their attributes (probability and money) can influence human risk preferences. Considering the evidence which indicates that people’s risk preferences can be guided by comparisons of gambles’ attributes, this is surprising, as gamble presentation has the potential to influence the ease with which attribute comparisons can be conducted. Accordingly, in this article, we will explore the influence of gamble presentation on peoples’ risky choice preferences.

Our proposal that the presentation of risky gambles can influence human risk preferences even when the content of the gambles is static is not implausible. For instance, in a similar vein to our proposal, research exploring human non-risky preferences has revealed that the order in which stimuli are presented (and processed) influences people’s judgments and choices about the stimuli. For example, people tend to prefer consumer goods which are presented to them first (e.g., [24,25]) and are more likely to vote for someone if their name appears first on a ballot (see [26]). Likewise, peoples’ impressions of other individuals (e.g., [27,28]; for a review, see [29]) and groups [30] are influenced by the way in which information is presented. Specifically, humans are influenced by an anchoring effect (e.g., [31]) and are therefore disproportionately influenced by early information. For instance, in a famous example, Tversky and Kahneman [31] demonstrated that people’s estimates about the percentage of African nations in the UN can be influenced by spinning a wheel of fortune. In particular, participants gave higher estimates after witnessing that the wheel of fortune landed on a high number (e.g., 65) rather than a low number (e.g., 10), and lower estimates when the wheel of fortune landed on a low number (10) rather than a high number (65).

Within the context of risky choice, a number of studies have explored the influence of presentation effects on human psychological processing and preferences (for a review, see [32]). For example, in an eye-tracking study which explored predictions derived from prominent theories of risky choice, Glöckner and Herbold [33] elicited participants’ risk preferences using a novel presentation format where the gambles attributes were presented in an ellipsoid display. When the results were compared to an identical study [34] that used a line-presentation format where the gamble’s attributes were presented in a line, Glöckner and Herbold’s results revealed slower decision-making time with the ellipsoid display. However, regardless of presentation type (ellipsoid or line), participants demonstrated a similar pattern of preferences. Furthermore, Franco-Watkins and Johnson [35] examined the extent to which different methods of preference elicitation and measurement impact people’s risky choice behavior. In particular, they focused on mouse tracking and eye tracking. With mouse tracking, attribute information is revealed when the mouse cursor is moved over the specific location associated with the information. In contrast, with eye tracking, information is not withheld; it does not need to be revealed by moving a cursor. The results indicate that decision-making time is greater for people measured using mouse tracking than eye tracking. Moreover, participants recorded via mouse tracking appear to choose riskier gambles. Taken together, these findings indicate that the perceptual properties of presented information do impact people’s risky behavior. More recent empirical evidence that perceptual presentation can impact cognitive processing comes from Liu et al. [36], who demonstrated that during choice between risky gambles, people make more utilitarian (expected value maximizing) choices when the presentation of the gambles encourages evaluation between alternatives, rather than the evaluation of specific attributes.

### 1.2. Age-Related Changes in Decision-Making

Outside of exploring context, behavioral science researchers are also concerned with investigating the influence of biological and social factors, such as age and aging, on risky decision-making behavior (e.g., [6]). Indeed, empirical evidence indicates that older and younger people demonstrated different patterns of risky judgments and behaviors, across a variety of risk domains. For example, results from a survey study conducted by Rhodes and Pivik [37] revealed that teen drivers perceived driving to be a less risky activity than adult drivers. Furthermore, when compared to adult drivers, teen drivers also self-reported that they engage in riskier driving behavior, such as driving faster than the speed limit and racing other cars, and found such behaviors more enjoyable. In the domain of public health, Rosi et al. [38] explored the risk perception of adults aged 18 to 87 towards COVID-19. The results revealed that while the perceived vulnerability of catching COVID-19 decreased as a function of age (i.e., older people judged themselves as less likely to contract the illness), the perceived severity of COVID-19 increased with age (i.e., older people judged the severity of illness as greater than younger people). Moreover, Bonem et al. [39] provided further evidence that the relationship between age and risky behavior varies across domains. Specifically, Bonem and colleagues’ research revealed that when compared to younger adults, older adults rated health and safety risks (e.g., regularly eating unhealthy foods) as more risky and judged themselves as less likely to engage in health and safety risks. In contrast, in the social domain, this pattern was reversed; compared to younger adults, older adults perceived less risk and judged themselves as more likely to engage in socially risky behavior (e.g., going to a social event by themselves).

In the domain of economic risk, the results are mixed. For instance, Kovalchik et al. [40] empirically explored the risky behavior of older (ages 70–95) and younger (ages 18–26) participants in the Iowa gambling task. Their results revealed that the two groups performed similarly; there were no statistically significant differences between the older and younger participants. In contrast, in a financial investment task, Samanez-Larkin et al. [41] reported that older adults made more suboptimal choices than younger adults. Specifically, during a task that required participants (aged between 19 and 85) to make 100 choices between financial options (stocks and bonds), older adults more frequently chose non-utility-maximizing options. Furthermore, in the domain of gain, during choice between a risky and a certain gamble, Mather et al. [42] demonstrated that when compared to younger adults, older adults were more risk-averse, as they had greater preference for a smaller but certain gain than a larger but risky (uncertain) gain. Likewise, in the domain of loss, older adults preferred a smaller but certain loss over an uncertain larger loss. Therefore, Mather and colleagues found that older adults have a greater preference for certainty than younger adults. Similarly, risk-averse tendencies among older adults in the context of economic risk were reported by Albert and Duffy [43], who found that during repeated choice between lotteries varying in risk and reward, older adults had greater preference than younger adults for safer gambles and demonstrated greater temporal discounting, resulting in more frequent non-utility-maximizing choices. In contrast, research conducted by Sparrow and Spaniol [44] found that older adults are not always more risk-averse than younger adults, and their ability to delay gratification often surpasses that of younger individuals. Moreover, their study revealed that aging is linked with structural, functional, and neurochemical changes in the brain that influence decision-making. Accordingly, Sparrow and Spaniol concluded that the impact of aging on decision-making is complex and influenced by cognitive, motivational, and social factors.

To account for reported differences in behavior between younger and older adults, it has been argued that factors such as cognitive aging, physical aging, lifestyle changes, intelligence (crystallized and fluid), experience, and life goals influence the decision-making of older adults. For example, de Acedo Lizárraga et al. [45] found that when making decisions, in contrast to young adults (aged 18 to 25), older people (aged 26 to 65) are less concerned with emotions and social pressure. Moreover, older adults weigh their decisions globally, taking into consideration factors like money constraints, goals, and work pressure.

A popular construct used in the exploration of cognitive aging is reaction time and processing speed [46]. In particular, when compared to younger adults, older adults tend to have a slower reaction time (e.g., [47,48]) and also tend to be slower at processing information [46,49,50], which impacts risky behavior. Moreover, Koscielniak et al. [51] revealed that processing speed influences females’ risky behavior during the Balloon Analog Risk Task (BART). Specifically, Koscielniak and colleagues demonstrated that older adults were less risk tolerant (more risk-averse) than younger adults even in situations where risk-seeking would have been a more effective (utilitarian) strategy.

Due to the decreased speed of cognitive processing [46,49] older people as opposed to younger people are more likely to make decisions informed by heuristic processing, rather than more computationally intense cognitive processes [52,53,54]. Overreliance on heuristics—psychological shortcuts—can negatively impact people’s ability to make normative rational (utility-maximizing) choices, increasing the likelihood of non-utility-maximizing choices. For example, across various risky domains (e.g., selection of health insurance or selection of retirement saving plans), Besedeš et al. [55] empirically demonstrated that older people rely on heuristics more than younger people and make fewer utility-maximizing decisions. Moreover, as reported by Mather et al. [42], older people are more dismissive of expected value than younger people. Although heuristics are typically framed as a cause of bias (e.g., [56]), Worthy et al. [57] argued that they help older adults to preserve decision quality under cognitively taxing circumstances (i.e., satisficing).

Furthermore, it has been argued that older adults compensate for their use of heuristic process, by leaning more heavily on their existing knowledge and past experiences (e.g., [45,58,59]). This existing knowledge and experience have the potential to influence older people’s interpretation of probabilistic information, leading to more risk-averse behavior [60,61] Although, risk taking behavior has been documented [62]. Furthermore, although slower reaction times and prolonged decision-making processes observed in older individuals may be attributed to biological changes, we propose that the extended decision-making duration can also be linked to their existing extensive knowledge and reliance on experience. Consequently, given their wealth of knowledge and experience, it is plausible that older participants will require more time than their younger counterparts when making utilitarian errors.

Our proposal that experience contributes to slower reaction times amongst older adults, especially when making utilitarian errors, is consistent with existing research (e.g., [63,64,65,66]. For instance, Blanco et al. [63] suggested that slower decision-making amongst older adults is caused by knowledge effects rather than cognitive decline; as knowledge increases with experience, there is greater cognitive processing load, resulting in slower decisions (see also [66]). Indeed, consistent with this proposal, in a series of exploratory choice task experiments, Blanco and colleagues’ [63] results indicated that experience does contribute towards accounting for differences in processing speed between younger and older adults. Moreover, research conducted by Eberhardt et al. [64] revealed that the higher levels of experience-based knowledge reported by older adults benefited their financial decision-making (e.g., credit card repayment decisions, money management, etc.). In particular, Eberhardt and colleagues [64] reported that older adults outperformed younger adults on financial decisions related to common tasks, where they are able to take advantage of their experience-based knowledge.

## 2. Method

### 2.1. Participants

All participants were recruited via an online data panel service and received a payment of GBP 1 for their participation. A window of 14 days was set for data collection, and by the end of this window, 278 participants had completed the experiment. The mean age of participants was 53 years old (*M* = 52.71, *SD* = 12.89) and 137 (50.5%) of the participants identified as female.

A significance level of 0.05 was used for statistical testing. In this experiment, an effect size was not assumed. However, a retrospective power analysis was employed to determine whether the sample size allowed for the detection of a large effect size (f = 0.40 by convention; [67]) of the independent-measures effects of *type of gamble presentation*, *median age*, and their interaction. A large effect size will achieve a statistical power of at least 0.95. Post hoc power analysis demonstrated that the sample size (N = 278) produced a power of 0.99, which exceed the target of 0.95.

The experiment received approval from the University of Huddersfield Business School’s research ethics committee. Moreover, all participants were treated in accordance with the British Psychological Society’s code of ethics and conduct, and the American Psychological Association’s ethical principles.

### 2.2. Experimental Design

A 2 × 2 independent measures design was used, with the following independent variables: (i) type of gamble presentation (horizontal or vertical) and median age (below and including 52 or above 52). The dependent variable was the participants’ utilitarian behavior; a binary choice between a utilitarian gamble offering a higher expected value (GBP 0.275) and a non-utilitarian gamble offering a lower expected value (GBP 0.225).

### 2.3. Materials and Procedure

To access the experiment, participants clicked on a website link which directed them to the study. Upon entering the study, participants read an information sheet which provided general information about the experiment. For example, about the data that would be collected, how it would be used, how it would be stored, and how they could withdraw from the study. After reading the information sheet, participants completed a consent form. Crucially, only participants who consented were able to take part in the experiment; participants who did not consent were directed out of the study and thanked for their time.

The participants who did provide consent and entered the experiment were first asked to provide basic demographic information (age and gender identity). Then, they were pseudo-randomly allocated to one of four experimental conditions: (i) type of gamble presentation (horizontal) × median age (below and including 52); (ii) type of gamble presentation (horizontal) × median age (above 52); (iii) type of gamble presentation (vertical) × median age (below and including 52); or (iv) type of gamble presentation (vertical) × median age (above 52). In all experimental conditions, participants had to make a choice between a utilitarian gamble and a non-utilitarian gamble:

*‘Choose one of the following two hypothetical options. For each of the options (A and B), the probability (%) represents the chance of winning the amount of money (£).’*

Specifically, participants had to choose between a utilitarian gamble offering a higher expected value (GBP 0.275) and a non-utilitarian gamble offering a lower expected value (GBP 0.225). Accordingly, participants made a choice between a utilitarian gamble offering a 55% chance of winning GBP 0.50 (gamble A) and a gamble offering a 15% chance of winning GBP 1.50 (gamble B).

**Figure 1:**
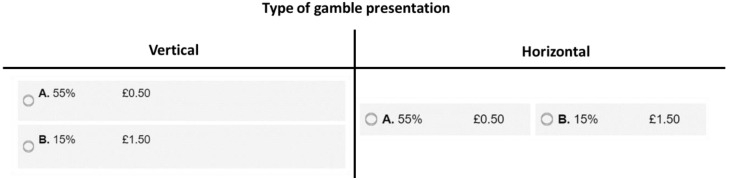
Gamble presentations.

The independent variable, type of gamble presentation, included two levels: (i) horizontal and (ii) vertical (see Figure 1). Accordingly, the presentation of the gambles was manipulated so that they were either presented horizontally side-by-side or vertically one above the other (see Figure 1).

After making their choice, participants were thanked for their time, directed out of the study, and subsequently financially compensated for their participation. The financial compensation was not performance-based; all participants were compensated equally as the choice task was hypothetical.

According to the methodological conventions of behavioral economics, the provision of incentives (e.g., performance-based financial rewards) is regarded as necessary for motivating research participants to carefully consider the decision-making task and to perform well (see [68,69]). In contrast, behavioral science research is often hypothetical and unincentivized, as research participants are expected to have internal motivation to perform well [69]. Empirically, findings regarding the relationship between incentivization, risky behavior, and performance are mixed (for a review, see [69]). For example, Irwin et al. [70] reported that during an experiment that required participants to bid for insurance against a real or hypothetical low-probability financial loss, monetary rewards reduced variability in participants’ bids for insurance and brought their bids closer to optimality. In contrast, it has also been argued and demonstrated that financial incentives do not improve rational normative decision-making (e.g., see [71,72]). Although, regardless of whether, when, and to what extent financial incentives influence human risky decision-making and performance, Wakker [73] argued that hypothetical choice is central for normative applications and should not be ignored. Moreover, Camerer and Hogarth [68] claim that ‘no replicated study has made rationality violations disappear purely by raising incentives’ (p. 7) (see also [74]). Indeed, according to Jenkins et al. [75], the relationship between financial incentives and performance is weakest in laboratory experiments.

## 3. Results and Discussion

### 3.1. Risky Choice

A binary logistic regression was conducted with the predictors type of gamble presentation (horizontal or vertical), median age (below and including 52 or above 52), and their interaction. The outcome variable was the participants’ utilitarian behavior (utilitarian or non-utilitarian). The results revealed that the regression model was a significant fit to the data, $\chi^{2}$(3) = 14.01 and *p* = 0.003. Moreover, the only predictor that made a significant contribution to the model was the type of gamble presentation (odds ratio, OR EXP[B] = 0.34, CI[0.95] = [0.148; 0.795], *p* = 0.013), which was negatively associated with participants’ utilitarian behavior. Therefore, the other predictors, median age (odds ratio, OR EXP[B] = 1.06, CI[0.95] = [0.512; 0.2.172, *p* = 0.873) and the two-way interaction between the type of gamble presentation and median age (odds ratio, OR EXP[B] = 1.06, CI[0.95] = [0.331; 0.3.390], *p* = 0.924), were statistically non-significant. Further binary logistic regression analysis explored the relationship between age (as a continuous variable) and choice; however, as reported above, the results revealed a non-significant relationship, $\chi^{2}$(52) = 64.75 and *p* = 0.110.

**Figure 2:**
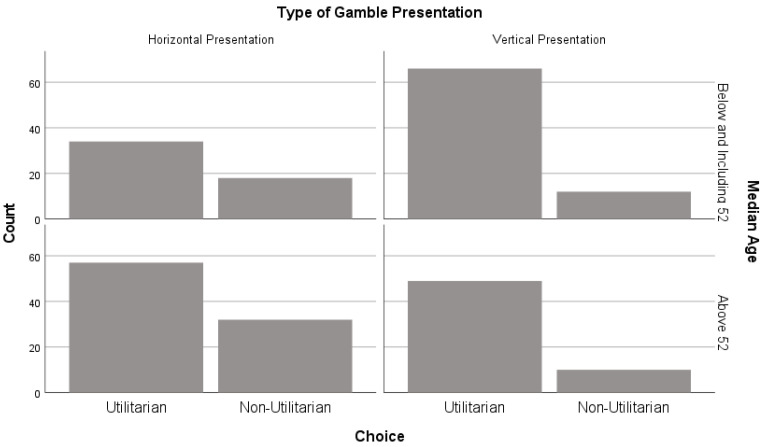
Participants’ utilitarian behavior by type of gamble presentation and median age.

Accordingly, the results revealed that the OR for choosing the non-utilitarian choice was 0.34 times smaller when the presentation of the gambles was vertical rather than horizontal (see Figure 2). In other words, the odds of participants choosing the non-utilitarian gamble were smaller with vertically presented gambles than with horizontally presented gambles (see Figure 2). Furthermore, this result indicates that participants’ utilitarian behavior was guided by ‘between gamble’ comparisons on the attributes available for comparison. Specifically, utilitarian choices (e.g., choosing Gamble A) were more commonly made when the gambles were presented vertically (easier comparison on the probability attribute, with Gamble A having a higher value) than when the gambles were presented horizontally (more difficult comparison on the probability attribute).

### 3.2. Decision-Making Time (Log Transformed)

A 2 × 2 × 2 ANOVA was conducted with the independent variables type of gamble presentation (horizontal or vertical), median age (below and including 52 or above 52), and choice (utilitarian and non-utilitarian) and the dependent variable decision-making time (log transformed [Ln]). The results revealed that the type of gamble presentation (*F* [1,270] = 0.08, *p* = 0.779), median age (*F* [1,270] = 0.05, *p* = 0.825), and choice (*F* [1,270] = 2.05, *p* = 0.154) did not have a statistically significant influence on participants’ decision-making time. Moreover, the two-way interactions median age by type of gamble presentation (*F* [1,270] = 2.31, *p* = 0.130), median age by choice (*F* [1,270] = 0.27, *p* = 0.603), and type of gamble presentation by choice (*F* [1,270] = 0.89, *p* = 0.346) had no statistically significant influence on decision-making time. Following the results reported in Section 3.1, we have explored with linear regression analysis the association between age (as a continuous variable) and decision-making time. Accordingly, the results revealed a non-significant relationship, *F*(1,276) = 0.16, *p* = 0.687, R = 0.024, and $R^{2}$ = 0.001.

**Figure 3:**
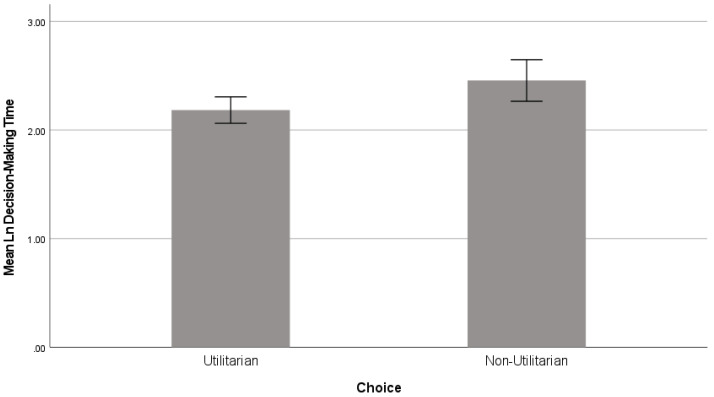
Participants’ decision-making time. Error bars represent 95% confidence intervals of the mean.

However, the three-way interaction of median age by type of gamble presentation by choice did significantly influence participants’ decision-making time, *F*(1,270) = 4.58 and *p* = 0.033, with a small effect size $\eta_{p}^{2}$ = 0.017. In particular, when the file was split by age to include only those aged above the median of the sample (52 years), a follow-up independent samples t-test revealed a statistically significant difference between the time taken to choose between the utilitarian (*M* = 2.18 and *SD* = 0.63) and non-utilitarian (*M* = 2.46 and *SD* = 0.61) gambles, *t*(146) = −2.38, *p* = 0.018, and *d* = −0.43 (see Figure 3). These results indicate that participants aged over 52 took more time to choose the non-utilitarian gamble than the participants aged 52 or younger. As we argued, and according to existing research (e.g., [50]), older people take longer to make decisions. Accordingly, as we proposed, it is plausible that making utilitarian decision errors takes older participants more time than younger participants, due to their existing knowledge and experience.

## 4. Conclusions

No previously published behavioral science research has investigated the influence of gamble presentation (horizontal or vertical) on people’s utilitarian behavior. This is surprising given evidence suggesting that individuals’ utilitarian behavior can be influenced by comparing attributes (e.g., probability and money) of gambles. Therefore, this article sought to explore the influence of gamble presentation on individuals’ utilitarian behavior. The results revealed that the likelihood of participants choosing the non-utilitarian gamble was lower with vertically presented gambles compared to horizontally presented gambles. Accordingly, we found that participants’ choices were influenced by between-gamble comparisons of the available attributes. Specifically, utilitarian choices (e.g., choosing Gamble A) were more prevalent when gambles were presented vertically than horizontally. This is because with vertical presentation, the comparison on the probability attribute was straightforward; crucially, Gamble A had a greater value on the probability attribute, and it was also the utilitarian option that participants commonly selected.

Additionally, published research indicates that older adults have a slower reaction time and take longer to process information (e.g., [50]). However, in this article, we proposed that due to their wealth of knowledge and experience, older participants would require more time than their younger counterparts when making utilitarian errors. Consistent with this proposal, the results revealed that participants aged over 52 took more time to choose the non-utilitarian gamble compared to those aged 52 or younger.

More broadly, the empirical results of this article pose a methodological challenge to risky judgment and decision-making studies that have asked people to choose between two gambles, but which have not controlled for the influence of presentation (horizontal and vertical). Within the context of risk, the choice between probabilistically defined gambles has been an indispensable tool for eliciting people’s risk preferences [76]. Indeed, they have been used to validate seminal theories of risky decision-making, such as Prospect Theory [1,3] and Decision by Sampling [13]. However, as existing behavioral theories have not accounted for presentation in their modeling or experimental method (design, materials, and procedure), it is possible that their results and conclusions might be confounded by presentation influences. Therefore, future studies which explore risky judgement and decision-making using gambles (or other tasks with probabilistically defined information) should control for the influence of presentation in their design. The most obvious way to achieve this is by counterbalancing the presentation of gambles, half with horizontal presentation and half with vertical presentation.

Of course, the finding that gamble presentation impacts people’s utilitarian behavior in the domain of risk has potential ‘real-world’ implications. For instance, the results imply that people’s decisions for risky products (e.g., financial products, insurance contracts, and gambling) could be influenced by how those products are presented. In particular, an organization could manipulate the presentation of products attributes which relate to risk and reward and influence peoples’ risky choices in the direction of the option dominant on the easiest to compare attribute. Moreover, decision support tools such as aggregator websites would also benefit from taking into consideration the results from this study, specifically, as the approach they use to present attribute information (e.g., price) has the potential to influence their users’ choice behavior. As aggregator websites are widely used by people to compare providers for a range of services (e.g., insurance, finance, and utilities), this could have significant financial and risk-related implications.

## Data Availability

Data are available upon request from the corresponding author (Joseph Teal—tealj@lsbu.ac.uk).
